# A Comparative Study of Coagulation Profiles in Preeclamptic and Normotensive Patients in Relation to Maternal and Fetal Outcomes

**DOI:** 10.7759/cureus.67940

**Published:** 2024-08-27

**Authors:** Hemant G Deshpande, Urvashi R Jainani, Ananya R Kiran, Shrestha Saha, Hada V Vanrajsinh

**Affiliations:** 1 Obstetrics and Gynecology, Dr. D Y Patil Medical College, Hospital and Research Centre, Dr. D Y Patil Vidyapeeth (Deemed to be University) Pimpri, Pune, IND

**Keywords:** normotensive, fetal outcome, maternal outcome, pre-eclampsia, coagulation

## Abstract

Background

Hypertensive complications during pregnancy play a significant role in the increased rates of maternal and perinatal morbidity and mortality on a global scale. Preeclampsia is characterized by elevated blood pressure levels and the presence of protein in the urine and is associated with diverse hematological alterations, particularly impacting the coagulation cascade. The primary objective of this research was to conduct a comparative analysis of the coagulation profiles and pregnancy outcomes in women with preeclampsia versus those with normal blood pressure during pregnancy.

Methods

This was a prospective case-control study with 74 participants across two groups, conducted from September 2022 to May 2024. The participants were enrolled and divided into two groups, with 37 in the clinically diagnosed preeclampsia group and 37 in the normotensive group. Coagulation parameters including platelet count, bleeding time, clotting time, international normalized ratio (INR), activated partial thromboplastin time (APTT), prothrombin time (PT), fibrinogen levels, alkaline phosphatase (ALP) levels, D-dimer levels, and fibrin degradation products (FDP) levels were assessed. Maternal and neonatal outcomes were also compared. In our study, we comprehensively examined both maternal and neonatal outcomes in preeclampsia and normotensive groups. Maternal complications analyzed included mode of delivery, incidence of eclampsia, placental abruption, hemolysis, elevated liver enzymes, and low platelets (HELLP) syndrome, postpartum hemorrhage (PPH), and peripartum cardiomyopathy (PPCM). For neonatal outcomes, we assessed birth weight, appearance, pulse, grimace, activity, and respiration (APGAR) scores, and the duration of neonatal intensive care unit (NICU) stays.

Results

The results showed that mean platelet count was significantly lower in the preeclampsia group (151,503 ± 59,875/µL) compared to the normotensive group (245,405 ± 69,021/µL) (p < 0.0001). Bleeding time, INR, APTT, and PT showed significant elevation in the preeclampsia group, indicating a slower coagulation process. Fibrinogen levels, ALP levels, and D-dimer levels were significantly higher in the preeclampsia group (p < 0.0001). The preeclampsia group had a higher rate of cesarean sections (65% vs. 24%) and lower neonatal birth weights (mean 2.3 kg vs. 2.5 kg). APGAR scores were comparable between groups, but a higher number of neonates went to the NICU in the preeclampsia group (64.9% vs. 10.8%). The preeclampsia group also showed higher rates of low birth weight (27%), intrauterine growth restriction (27%), respiratory distress syndrome (10.8%), and asphyxia (5.4%).

Conclusion

Preeclampsia is associated with significant hematological changes, particularly in coagulation parameters, and adverse fetomaternal outcomes. Early identification and monitoring of these changes are crucial for timely intervention and improving maternal and neonatal health outcomes.

## Introduction

Hypertensive disorders in pregnancy (HDP) pose a significant global health burden, contributing to severe morbidity, long-term disability, and maternal deaths. These disorders account for about 14% of all maternal deaths globally [1]. HDP manifests through the initiation of elevated blood pressure occurring post-20 weeks of gestation in individuals who had normal blood pressure before this period. HDP affects approximately 6-8% of pregnancies. It is crucial to distinguish HDP from chronic hypertension, which is high blood pressure that existed before pregnancy or was diagnosed before 20 weeks of gestation [1,2]. Preeclampsia, a more severe form of HDP, involves high blood pressure and/or proteinuria (the presence of protein in the urine). It occurs in approximately 2-8% of pregnancies and is a leading cause of maternal and perinatal morbidity and mortality worldwide. Additionally, eclampsia, a severe complication of preeclampsia characterized by seizures, occurs in about one in 2000 pregnancies [1].

In India, the prevalence of hypertensive disorders in pregnancy varies by region. Overall, the prevalence is around 9.4%, with preeclampsia being the most common type, accounting for approximately 7.4% of cases [3]. These rates are notably higher than the global average, likely due to genetic predispositions, environmental factors, healthcare access, and quality disparities.

Hypertensive disorders of pregnancy can lead to various hematological changes, particularly affecting the coagulation system [4]. The coagulation cascade is typically activated in preeclampsia, resulting in a highly thrombotic and pro-coagulant state with platelet activation and thrombin and fibrin formation. Thrombocytopenia, or low platelet count, is the most common hematological abnormality in preeclampsia and eclampsia [5]. This condition can lead to a hypercoagulable state due to endothelial injury, a primary pathogenic factor in HDP [6]. The coagulation profile in patients with HDP often includes tests such as platelet count, bleeding time, clotting time, prothrombin time (PT), and activated partial thromboplastin time (APTT) [7]. These tests are crucial for the early assessment of coagulation abnormalities before complications like hemolysis, elevated liver enzymes, and low platelets (HELLP) syndrome, disseminated intravascular coagulation (DIC), and cerebrovascular complications occur [8]. In severe HDP cases, abnormalities in coagulation parameters like PT, APTT, and fibrinogen levels are observed. These abnormalities are prognostic markers and can serve as additional diagnostic criteria for preeclampsia, particularly in resource-limited settings [9].

Early identification and monitoring of hematological abnormalities are essential for timely therapy initiation and preventing severe complications. Accurate diagnostic criteria for preeclampsia are pivotal for effective management. This study aims to compare coagulation profiles in preeclampsia and normotensive patients to elucidate the differences and similarities between these groups. Understanding the coagulation changes in preeclampsia can inform preventive strategies and therapeutic interventions, reducing maternal and fetal morbidity and mortality. The results of this study also have the potential to strengthen antenatal care services for managing HDP complications.

## Materials and methods

Study design

This prospective study was carried out at Dr. D. Y. Patil Medical College, Hospital, and Research Centre in Pimpri, Pune, from September 2022 to May 2024. The internal ethics committee of the institute approved the study (IESC/PGS/2022/131). Informed written consent was taken from each participant. Participants were followed from recruitment through their pregnancy to observe the progression of coagulation parameters, providing insights into the management and outcomes of hypertensive disorders during pregnancy.

Sample size calculation

The sample size was determined using Winpepi software version 11.38, based on the mean and standard deviation of platelet counts in preeclampsia and normotensive pregnancy cohorts [10]. The calculation aimed to achieve 80% statistical power and a 95% confidence interval with a 5% significance level. The total sample size was determined to be 74, with 37 participants in each group.

Sampling technique

Convenience sampling was employed to recruit participants who met the inclusion criteria during their routine antenatal period. Investigations were sent at the time of admission at zero hours. Women who consented to participate were enrolled until the sample size of 74 was reached, with 37 participants in each group (normotensive and preeclampsia/eclampsia).

Participation criteria

The inclusion criteria for the study comprised four groups. The first group included women diagnosed with non-severe preeclampsia, characterized by elevated blood pressure (systolic blood pressure ≥140 mm Hg or diastolic blood pressure ≥90 mm Hg on two occasions at least four hours apart after 20 weeks of gestation in a previously normotensive woman) and proteinuria (300 mg or more per 24-hour urine collection, a urine protein/creatinine ratio of 0.3 mg/dL or more, a dipstick reading of 1+) without severe features. The second group encompassed women with severe preeclampsia, marked by significantly elevated blood pressure (systolic blood pressure of 160 mm Hg or higher, or diastolic blood pressure of 110 mm Hg or higher on two occasions at least four hours apart) and additional complications such as thrombocytopenia, impaired liver function, renal insufficiency, pulmonary edema, severe headaches, or visual disturbances. The third group included women with eclampsia, which involves pre-existing preeclampsia accompanied by convulsions that cannot be attributed to other causes. The first three groups were combined as 'preeclampsia' for further statistical analysis. The fourth group consisted of normotensive patients, defined as pregnant women with blood pressure ranging from 110/70 mm Hg to 130/90 mm Hg and no comorbidities related to hypertensive disorders of pregnancy [11].

Patients were excluded from the study if they had pre-existing medical disorders that could confound the results. These disorders included diabetes mellitus, renal disease, coagulopathies, chronic hypertension, hepatitis, placental abruption, or placenta previa. Additionally, women with a history of oral contraceptive use or severe trauma were also excluded from the study. These exclusion criteria ensured that the study population was homogeneous and that the observed effects could be attributed more confidently to hypertensive disorders of pregnancy.

Data collection

Demographic details were recorded upon enrollment. Detailed medical histories, clinical parameters, obstetric examinations, and coagulation profiles were assessed following standard guidelines. Maternal and fetal outcomes were documented systematically. Blood pressure measurements were taken using standardized techniques in both outpatient and inpatient settings.

Data analysis

Data was organized in Microsoft Excel (Microsoft Corporation, Redmond, Washington, United States) and analyzed using GraphPad Prism software. A t-test was used to compare the difference in mean values of the parameters. A P-value < 0.05 was considered statistically significant. 

## Results

A total of 74 participants were enrolled, with 37 in each group. In the preeclampsia group (n=37), the average maternal age was 26.3 years (±5), with a median age of 25 years, ranging from 20 to 40 years. The normotensive group had a slightly lower mean maternal age of 25.6 years (± 4.8), also with a median of 25 years and an age range of 20 to 38 years. In the preeclampsia group, the maternal age distribution showed that among 37 participants, five participants (13.5%) were in the 20-24 year range, four participants (10.8%) were in the 25-29 year range, 13 participants (35.1%) were in the 30-34 year range, and 15 participants (40.5%) were in the 35-39 year range. In the normotensive group, among 37 participants, four participants (10.8%) were in the 20-24 year range, 13 participants (35.1%) were in the 25-29 year range, 13 participants (35.1%) were in the 30-34 year range, and seven participants (18.9%) were in the 35-39 year range (Table 1).

**Table 1:** Distribution of age Frequency represented as n(%)

Age range (years)	Preeclampsia group (n=37)	Normotensive group (n=37)
20-24	5 (13.5%)	4 (10.8%)
25-29	4 (10.8%)	13 (35.1%)
30-34	13 (35.1%)	13 (35.1%)
35-39	15 (40.5%)	7 (18.9%)

**Figure 1:**
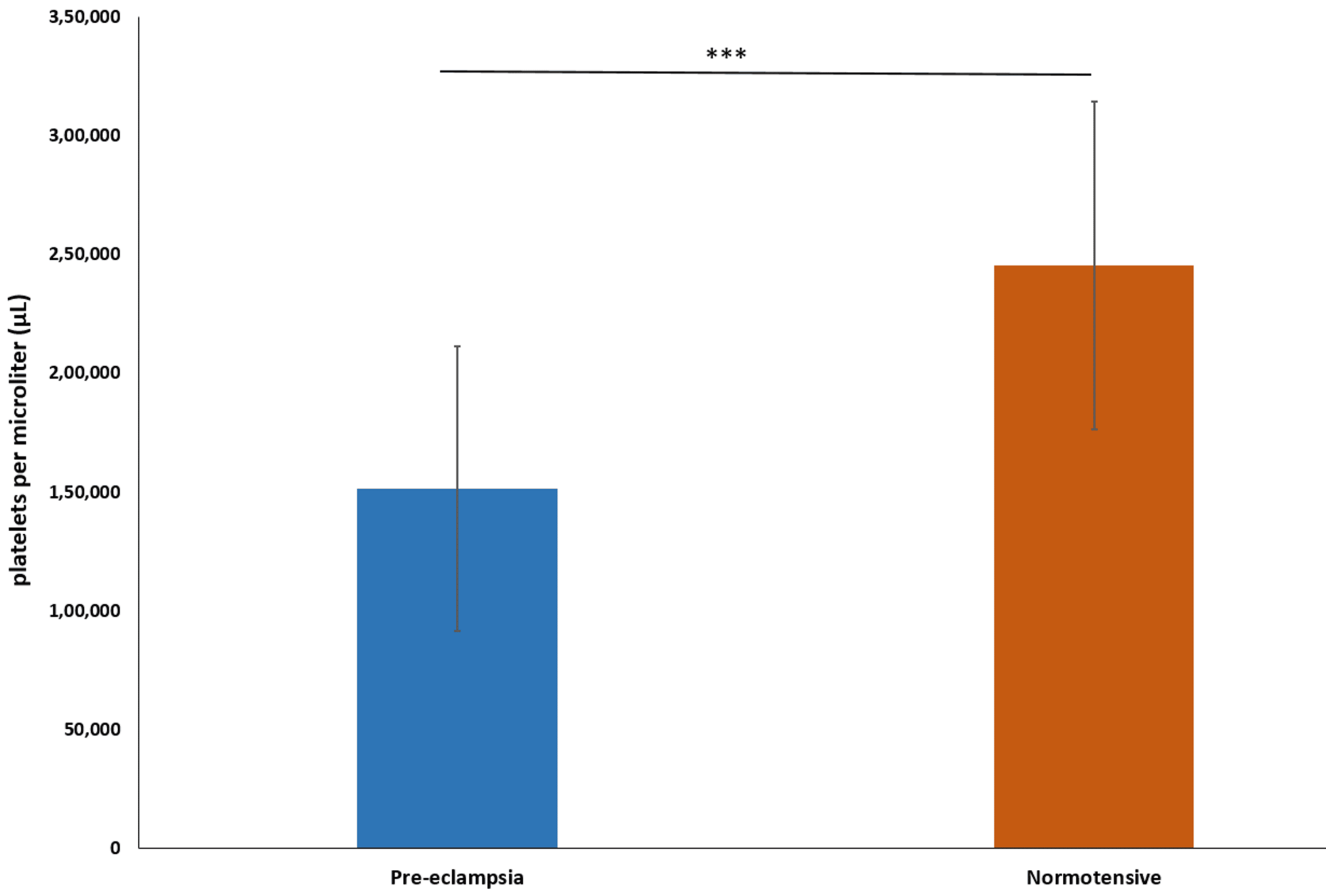
Bar graph representation comparison of mean platelet count *** indicates p-value<0.0001 calculated using t-test

The mean platelet count in the preeclampsia group was 151,503 ± 59,875 platelets per microliter (µL), with a median of 130,000 µL. In contrast, the normotensive group had a mean platelet count of 245,405 ± 69,021 µL, with a median of 276,000 µL. The difference was statistically significant (p-value < 0.0001) (Figure 1). 

**Figure 2:**
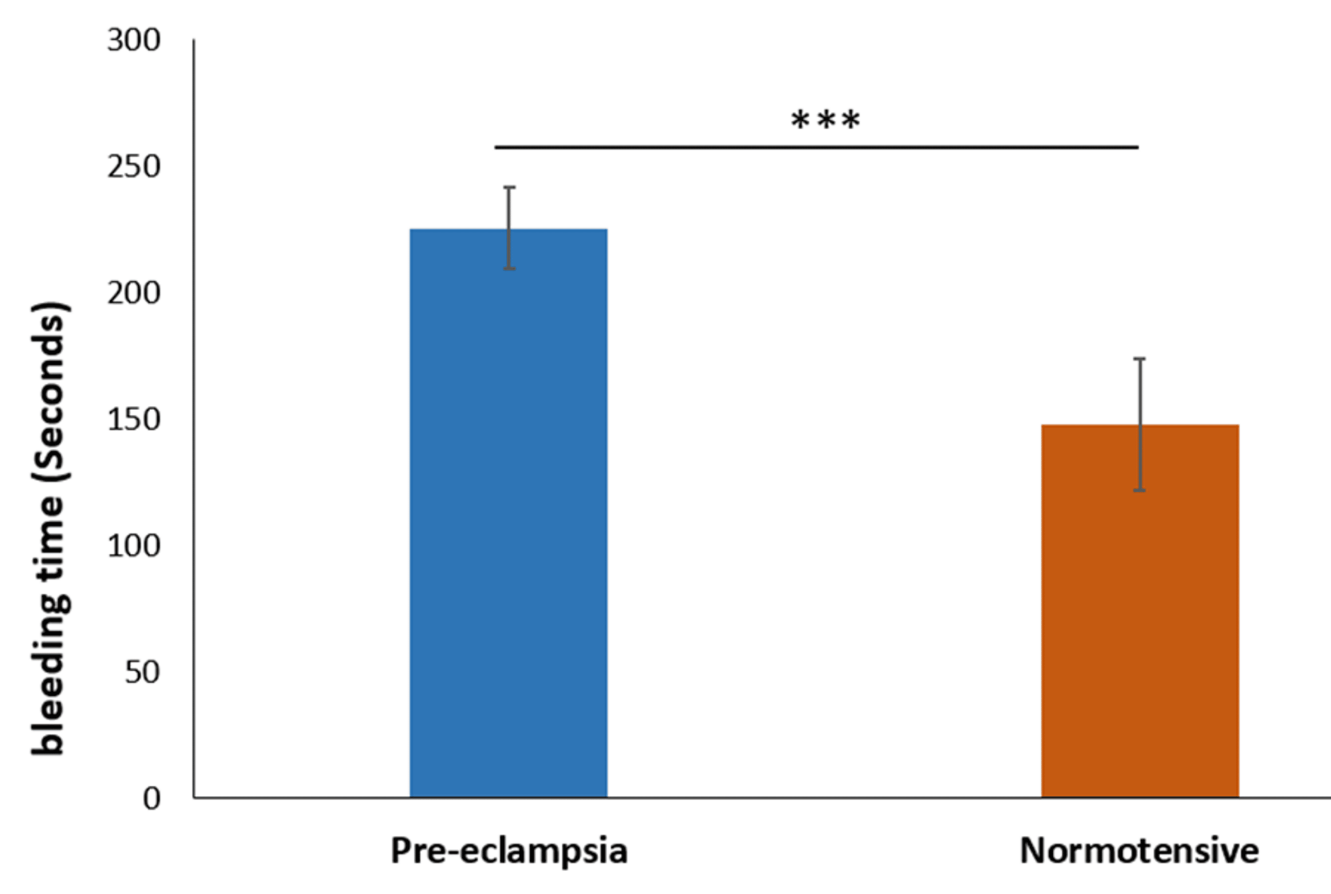
Comparison of bleeding time *** indicates p-value<0.0001 calculated using t-test

The mean bleeding time in the preeclampsia group was 225.5 seconds ± 16.13 seconds, with a median of 226.6 seconds. In contrast, the normotensive group had a mean bleeding time of 147.8 seconds ± 26.33 seconds, with a median of 149.4 seconds. The difference was statistically significant (p-value <0.0001) (Figure 2).

**Figure 3:**
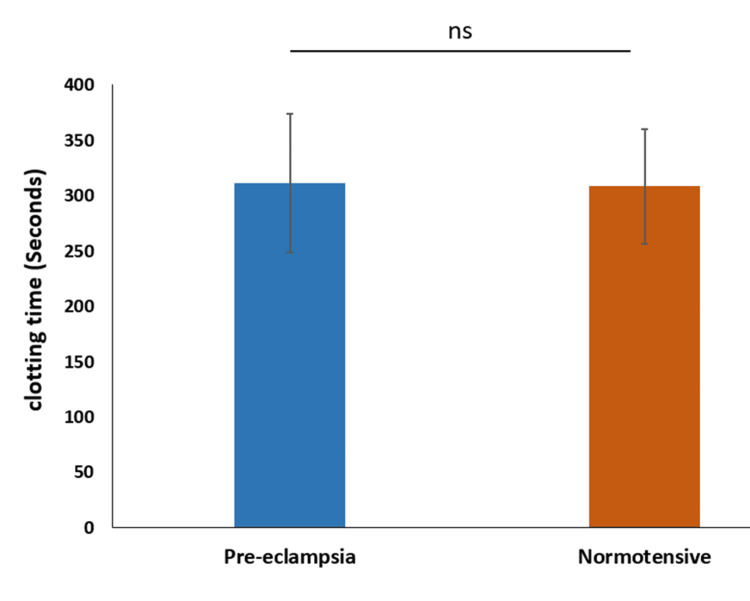
Comparison of clotting time ns indicates p-value>0.05, indicating non-significant. Calculated using t-test.

The mean clotting time in the preeclampsia group was 311.5 seconds ± 62.52 seconds, with a median of 318.7 seconds. In the normotensive group, the mean clotting time was 308.3 seconds ± 51.94 seconds, with a median of 309.0 seconds. These values suggest that the clotting times are relatively similar between the two groups, indicating no significant difference (p-value=0.811) in this parameter (Figure 3).

**Figure 4:**
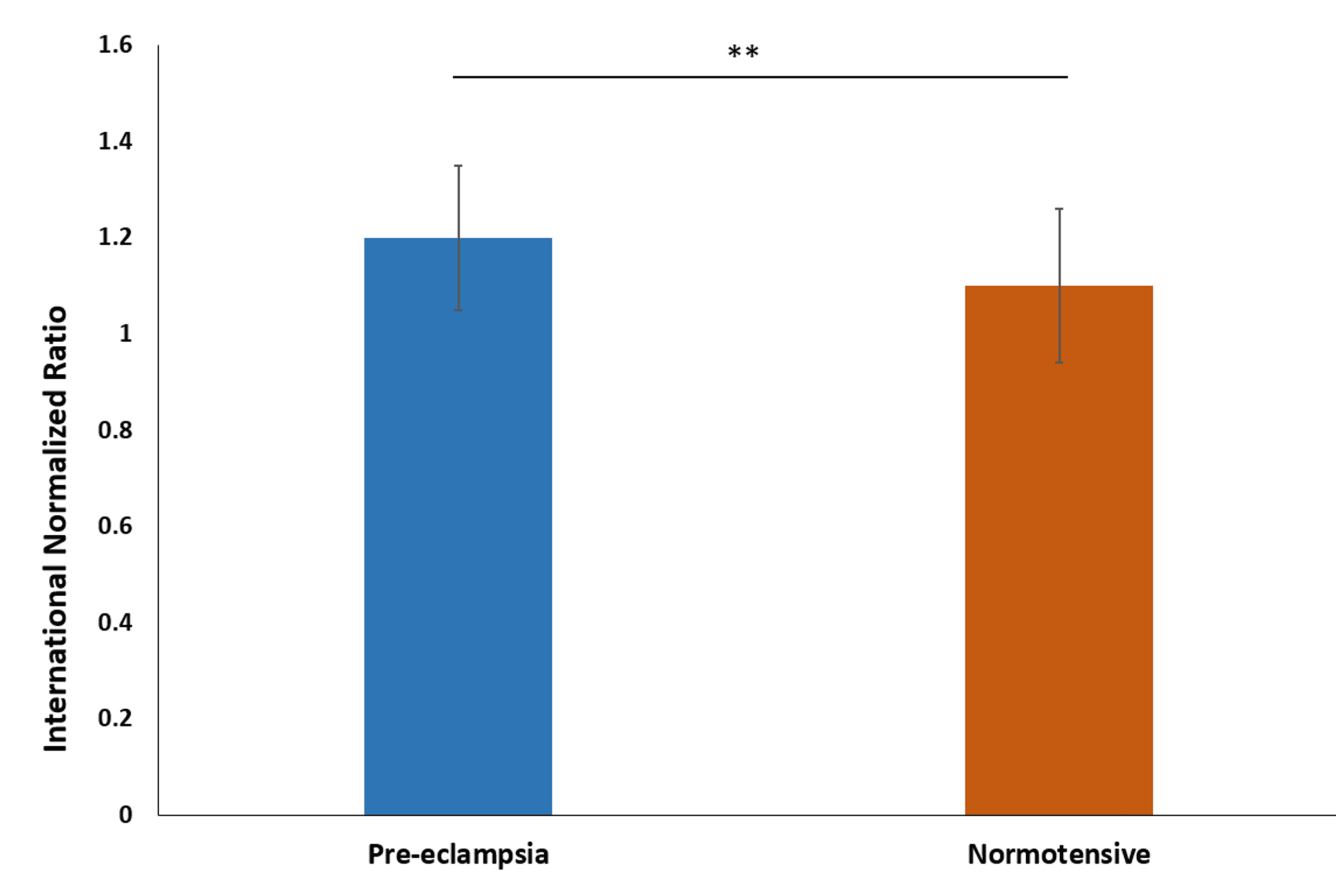
Comparison of international normalized ratio ** indicates p-value<0.001 calculated using t-test

The mean international normalized ratio (INR) in the preeclampsia group was 1.2 ± 0.16, with a median of 1.2. In the normotensive group, the mean INR was slightly lower, at 1.1 ± 0.154, with a median of 1.1. The INR in the preeclampsia group was significantly higher than the normotensive group (p-value=0.0089) (Figure 4).

**Figure 5:**
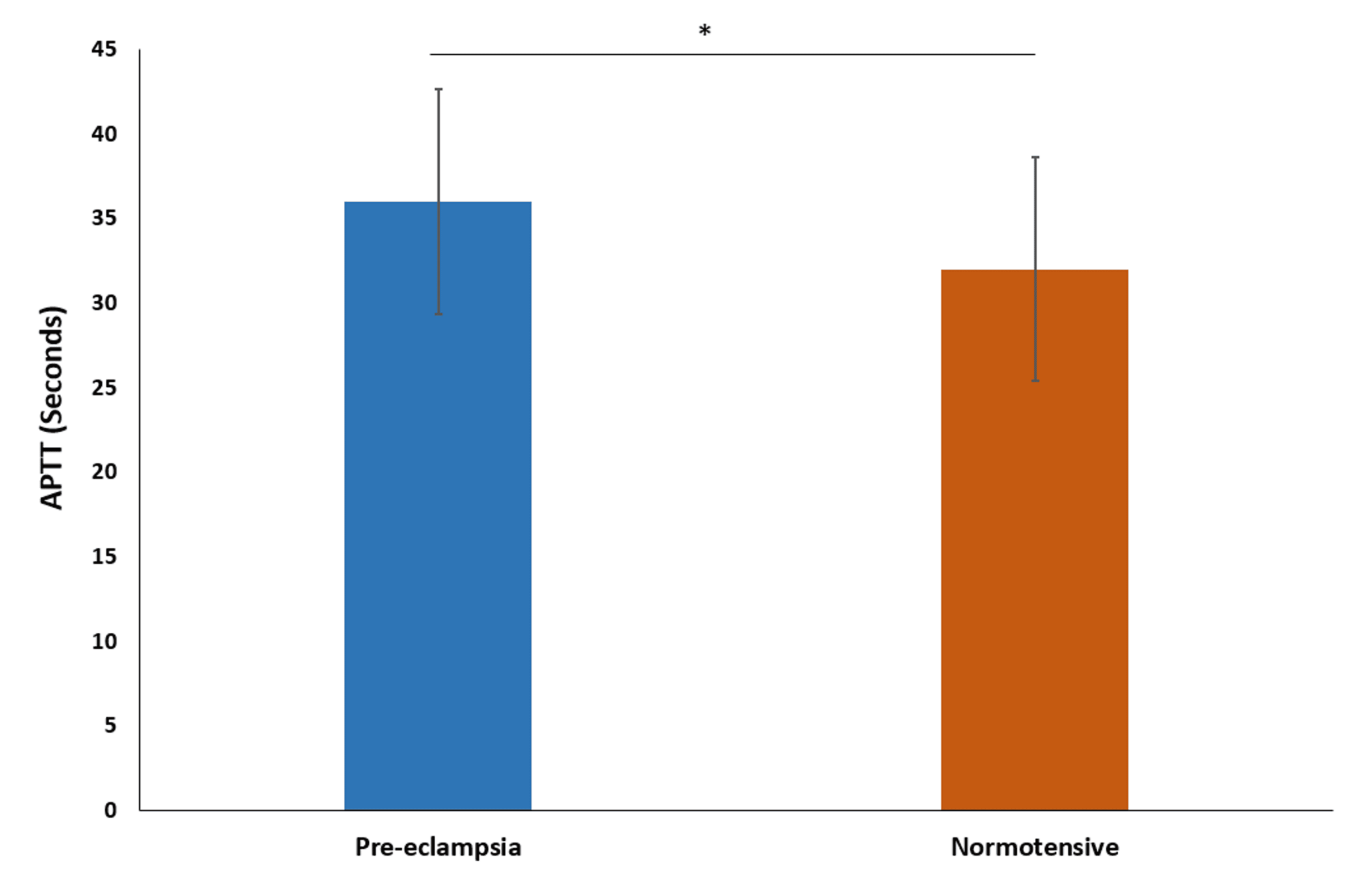
Comparison of activated partial thromboplastin time * indicates p-value<0.05 calculated using t-test

We further analyzed the activated partial thromboplastin time (APTT). The mean APTT in the preeclampsia group was 36.0 seconds ± 6.64, with a median of 36.1 seconds. In the normotensive group, the mean APTT was 32.0 seconds ± 6.60, with a median of 32.1 seconds. The APTT was significantly longer in the preeclampsia group (p-value=0.0113), suggesting a slower coagulation process compared to the normotensive group (Figure 5).

**Figure 6:**
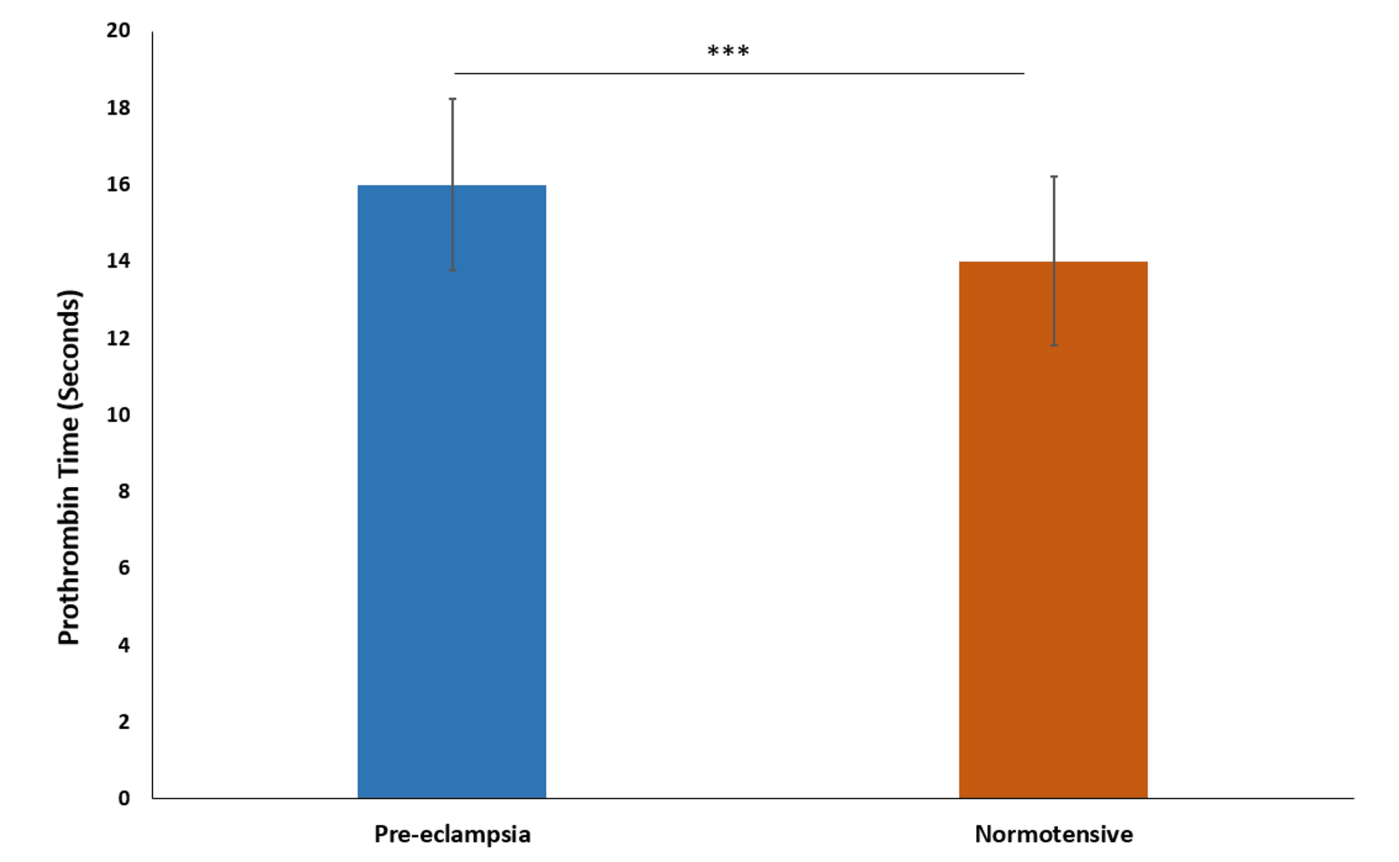
Comparison of prothrombin time *** indicates p-value<0.0001 calculated using t-test

The mean prothrombin time (PT) in the preeclampsia group was 16.0 seconds ± 2.23, with a median of 16.0 seconds. In the normotensive group, the mean PT was 14.0 seconds ± 2.20, with a median of 14.0 seconds. The PT is longer in the preeclampsia group, indicating a slower clotting time compared to the normotensive group (p-value=0.0002) (Figure 6).

**Figure 7:**
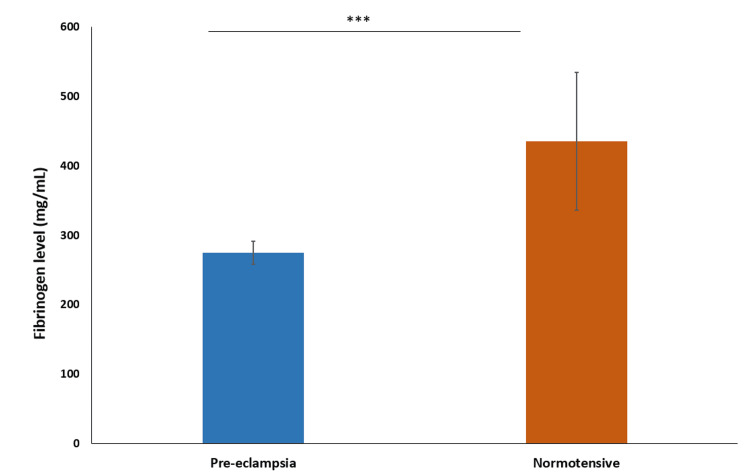
Comparison of fibrinogen level *** indicates p-value<0.0001 calculated using t-test

The mean fibrinogen level in the preeclampsia group was 274.7 mg/dL ± 16.53, with a median of 275 mg/dL. For the normotensive group, the mean fibrinogen level was 435.4 mg/dL ± 99.23, with a median of 435 mg/dL. This shows that the fibrinogen level is significantly lower in the preeclampsia group, indicating reduced clotting potential (P < 0.0001) (Figure 7).

**Figure 8:**
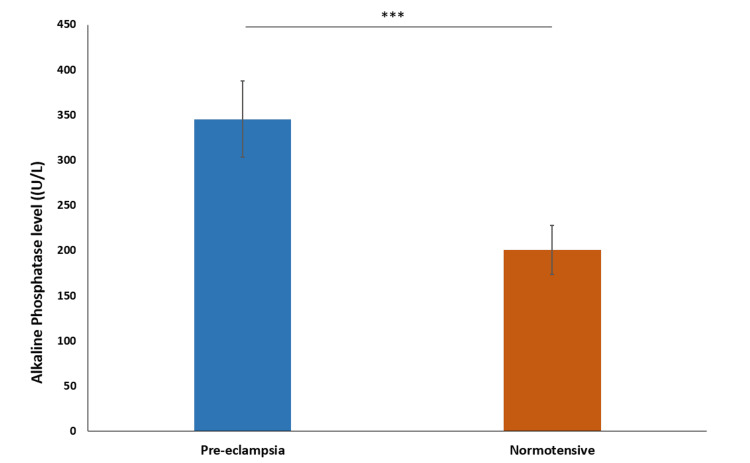
Comparison of alkaline phosphatase levels *** indicates p-value<0.0001 calculated using t-test

The mean alkaline phosphatase (ALP) level in the preeclampsia group was 345.5 U/L ± 42.17, with a median of 362 U/L. In the normotensive group, the mean ALP level was 200.6 U/L ± 27.04, with a median of 206 U/L. This suggests that the ALP level is significantly higher in the preeclampsia group (P < 0.0001) (Figure 8).

**Figure 9:**
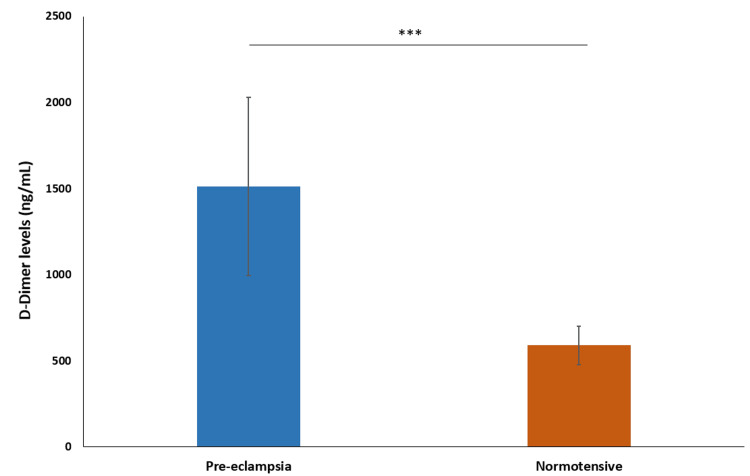
Comparison of D-dimer level *** indicates p-value<0.0001 calculated using t-test

The mean D-dimer level in the preeclampsia group was 1512.9 ng/mL ± 518.35, with a median of 1517 ng/mL. In the normotensive group, the mean D-dimer level was 589.8 ng/mL ± 110.60, with a median of 546 ng/mL. This shows that the D-dimer level is significantly higher in the preeclampsia group, indicating increased fibrinolysis and clot degradation (P < 0.0001) (Figure 9).

**Figure 10:**
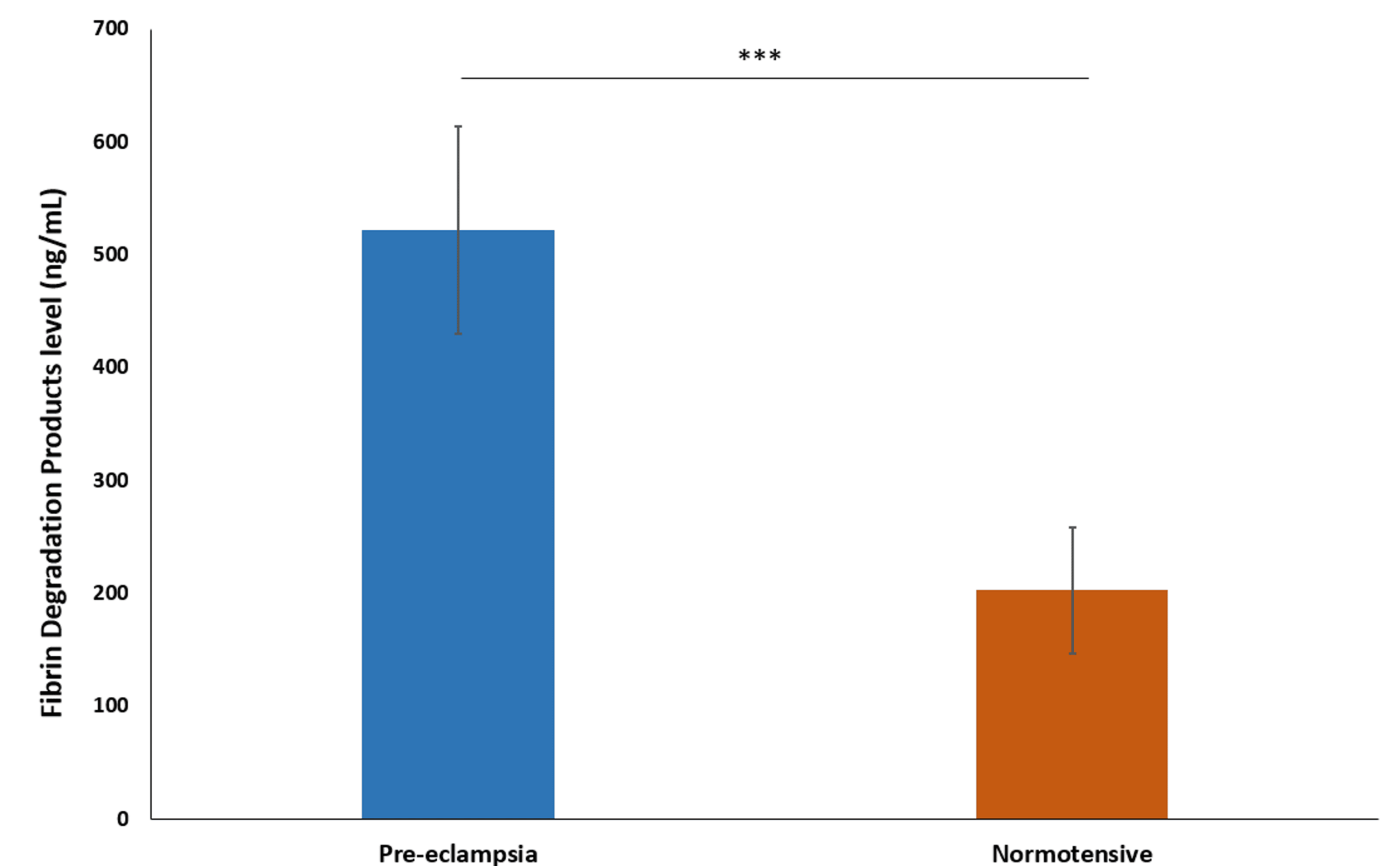
Comparison of fibrin degradation products level *** indicates p-value<0.0001 calculated using t-test

The mean fibrin degradation products (FDP) level in the preeclampsia group was 522.2 ng/mL ± 91.72, with a median of 500 ng/mL. For the normotensive group, the mean FDP level was 202.7 ng/mL ± 55.91, with a median of 212 ng/mL. This suggests that the FDP level is significantly higher in the preeclampsia group (P < 0.0001) (Figure 10).

In the preeclampsia group, 24 out of 37 participants (65%) were delivered by lower segment cesarean section (LSCS), while 13 participants (35%) were delivered full-term by normal vaginal delivery (NVD). In the normotensive group, nine out of 37 participants (24%) were delivered by lower segment cesarean section (LSCS), and 28 participants (76%) were delivered by NVD. This demonstrates a notable difference in delivery methods between the two groups, with a higher percentage of LSCS deliveries in the preeclampsia group and a higher percentage of full-term NVD deliveries in the normotensive group (Table 2).

**Table 2:** Maternal and neonatal outcomes Frequency represented as n(%) Mean represented as mean±standard deviation APGAR: appearance, pulse, grimace, activity and respiration; HELLP: hemolysis, elevated liver enzymes, and low platelets; NICU: neonatal intensive care unit

Category	Preeclampsia group (n=37)	Normotensive group (n=37)
Maternal outcomes		
Mode of delivery		
Lower segment cesarean section (LSCS)	24 (65%)	9 (24%)
Normal vaginal delivery (NVD)	13 (35%)	28 (76%)
Maternal complications		
Eclampsia	2 (5%)	1 (2%)
Placental abruption	3 (8%)	1 (2%)
HELLP syndrome	4 (10%)	0 (0%)
Postpartum hemorrhage (PPH)	6 (15%)	2 (5%)
Peripartum cardiomyopathy	3 (8%)	1 (2%)
Neonatal parameters		
Birth weight range (kg)		
>3.0	1 (2.7%)	7 (18.9%)
1.5 - 2.0	16 (43.2%)	6 (16.2%)
2.1 - 2.5	7 (18.9%)	6 (16.2%)
2.6 - 3.0	13 (35.1%)	18 (48.6%)
Mean APGAR score at 1 minute	6.7 ± 1.4	6.8 ± 1.4
Mean APGAR score at 5 minutes	8.2 ± 0.9	8.3 ± 0.9
Neonatal outcomes		
NICU Stay	24 (64.9%)	4 (10.8%)
Low birth weight (LBW)	10 (27%)	2 (5.4%)
Intrauterine growth restriction (IUGR)	10 (27%)	2 (5.4%)
Respiratory distress syndrome (RDS)	4 (10.8%)	0 (0%)
Asphyxia	2 (5.4%)	1 (2.7%)

The mean birth weight of the neonates in the preeclampsia group was 2.3 kg (±0.5), with a median of 2.4 kg, ranging from 1.5 to 3.0 kg. In the normotensive group, the mean birth weight of the neonates was slightly higher at 2.5 kg (±0.6), with a median of 2.6 kg, ranging from 1.5 to 3.3 kg. This showed slightly higher birth weights in the normotensive group. The analysis of birth weights revealed distinct patterns between the normotensive and preeclampsia groups. In the normotensive group (n=37), 18.9% (seven cases) had birth weights greater than 3 kg, 16.2% (six cases) had birth weights between 1.5 to 2 kg, 16.2% (six cases) had birth weights between 2.1 to 2.5 kg, and 48.6% (18 cases) had birth weights between 2.6 to 3 kg. In contrast, in the preeclampsia group (n=37), only 2.7% (one case) had birth weights greater than 3 kg, 43.2% (16 cases) had birth weights between 1.5 to 2 kg, 18.9% (seven cases) had birth weights between 2.1 to 2.5 kg, and 35.1% (13 cases) had birth weights between 2.6 to 3 kg (Table 2).

The mean APGAR score of the neonates at one minute in the preeclampsia group was 6.7 (±1.4), with a median of 7, ranging from 3 to 8. In the normotensive group, the mean APGAR score at one minute was slightly higher at 6.8 (±1.4), with a median of 7, ranging from 4 to 8. The mean APGAR score at five minutes in the preeclampsia group was 8.2 (±0.9), with a median of 9, ranging from 5 to 9. In the normotensive group, the mean APGAR score at five minutes was slightly higher at 8.3 (±0.9), with a median of 9, ranging from 6 to 9. In the preeclampsia group, 24 out of 37 newborns (64.9%) required a stay in the neonatal intensive care unit (NICU), while in the normotensive group, four out of 37 newborns (10.8%) required a stay in the NICU (Table 2).

We recorded various neonatal outcomes in the two groups, such as low birth weight (LBW), intrauterine growth restriction (IUGR), respiratory distress syndrome (RDS), and asphyxia. There were 10 cases (27%) of LBW, 10 cases (27%) of IUGR, and four cases (10.8%) of RDS in the preeclampsia group, indicating significant developmental and health complications in these newborns. Additionally, there were two cases (5.4%) of asphyxia in the preeclampsia group. In the normotensive group, neonatal outcomes were generally better, with fewer cases of LBW, IUGR, and RDS: two cases (5.4%) of LBW, two cases (5.4%) of IUGR, zero cases (0%) of RDS, and one case (2.7%) of asphyxia (Table 2). 

We further analyzed the maternal complications in our study population. In the preeclampsia group, the incidence of eclampsia was 5% (two cases), compared to 2% (one case) in the normotensive group. The incidence of placental abruption was 8% (three cases) in the preeclampsia group, compared to 2% (one case) in the normotensive group, indicating a greater risk of abruption in women with preeclampsia. The occurrence of HELLP syndrome was 10% (four cases) in the preeclampsia group, with no cases reported in the normotensive group. Postpartum hemorrhage (PPH) was observed in 15% (six cases) of the preeclampsia group, compared to 5% (two cases) in the normotensive group. Lastly, the incidence of peripartum cardiomyopathy was 8% (three cases) in the preeclampsia group, compared to 2% (one case) in the normotensive group. There were 23 cases (62.2%) of preeclampsia, and 20 cases (54.1%) of normotensive women (Table 2).

## Discussion

Our study identified a significant difference in platelet counts between the preeclampsia and normotensive groups, with the mean platelet count being notably lower in the preeclampsia group. This finding is consistent with the results reported by Walle et al. (2022), who also found significantly lower platelet counts in preeclampsia and eclampsia groups [10]. Similarly, Bhutani et al. (2022) reported significantly lower platelet counts in patients with gestational hypertension (201000 ± 54000/mm³) compared to normal patients (285000 ± 73000/mm³) [4]. Several other studies have shown lower platelet counts in preeclamptic patients [12-14].

Our investigation revealed significantly lower fibrinogen levels in the preeclampsia group compared to the normotensive group, suggesting a consumption coagulopathy. This aligns with the findings of Indora et al. (2022) and Priya et al. (2021), who also reported reduced fibrinogen levels in preeclamptic patients [15-17].

We observed a significant difference in the international normalized ratio (INR) between the preeclampsia and normotensive groups, indicating variations in blood clotting ability. Bhutani et al. (2022) reported significantly prolonged INR values in preeclampsia and eclampsia groups. Priya et al. (2021) found higher INR values in preeclampsia compared to the control group [4,16]. Lakshmi (2016) noted increased INR with the severity of preeclampsia, with values of 1.2 in mild preeclampsia, 1.3 in severe preeclampsia, and 1.4 in eclampsia also noted increased INR in preeclampsia [18].

The mean activated partial thromboplastin time (APTT) level was significantly higher in the preeclampsia group than in the normotensive group, indicating different blood clotting times between the groups. Priya et al. (2021) reported significantly prolonged APTT values in preeclampsia and eclampsia groups. Han et al. (2014) found prolonged APTT values in preeclampsia patients compared to the control group [19].

Our study also noted that the mean prothrombin time (PT) level in the preeclampsia group was significantly higher than in the normotensive group. Lefkou et al. (2020) reported significantly prolonged PT values in preeclampsia and eclampsia groups. They found prolonged PT values in preeclampsia (13.24 ± 0.80 seconds) to be higher compared to the control group (12.23 ± 0.59 seconds). Similar observations have been reported in several other studies [12,15,20]. 

The mean fibrinogen level in the preeclampsia group was significantly lower than in the normotensive group, highlighting different fibrinogen levels between the groups. Similar trends were observed by Lakshmi (2016), Sharma et al. (2016), Joshi et al. (2015), and Khan et al. (2018) [18,21-23].

Our study found significantly elevated D-dimer levels in the preeclampsia group compared to the normotensive group, indicating higher coagulation activation. Sharma et al. (2016) and Joshi et al. (2015) reported similar findings [21,22].

We observed significant differences in bleeding time and clotting time between the preeclampsia and normotensive groups. The mean bleeding time was longer in the preeclampsia group, suggesting impaired platelet function and vascular integrity. This finding is consistent with studies by Priya et al. (2021) and Sharma et al. (2016), which reported prolonged bleeding time in preeclamptic patients. Monitoring bleeding and clotting times in preeclamptic patients is crucial for early detection and management of potential coagulopathies. These parameters provide valuable insights into the hemostatic status of patients and guide clinical interventions to prevent complications such as disseminated intravascular coagulation (DIC) and severe hemorrhage. The significant prolongation of bleeding and clotting times in our study underscores the need for vigilant monitoring and comprehensive management of coagulation status in preeclampsia to improve maternal and fetal outcomes.

Ultrasound findings revealed significant differences between the preeclampsia and normotensive groups, with higher incidences of fetal growth restriction (FGR), oligohydramnios, and abnormal Doppler findings in the preeclampsia group. These results are consistent with those reported by Priya et al. (2021), who found similar adverse fetal outcomes associated with preeclampsia [16].

In terms of delivery modes, our study found that 65% of the preeclampsia group underwent lower segment cesarean section (LSCS) and 35% had full-term normal delivery (FTND). In contrast, 24% of the normotensive group underwent LSCS and 76% had FTND, indicating a significant difference in delivery modes between the groups, with a higher proportion of LSCS in the preeclampsia group. Studies by Sanchez et al. (2021) and Pasokpuckdee et al. (2023) also report higher rates of cesarean deliveries in preeclampsia groups [24,25].

Neonatal outcomes in our study showed significantly higher incidences of low birth weight (LBW), intrauterine growth restriction (IUGR), and respiratory distress syndrome (RDS) in the preeclampsia group. While most referenced studies did not focus on neonatal outcomes, Indora et al. (2022) reported significantly higher NICU admissions in the preeclampsia and eclampsia groups compared to the normotensive group, aligning with our findings of poorer neonatal outcomes in preeclampsia [15]. Other studies by Sharma et al. (2016) and Lakshmi (2016) noted higher incidences of adverse neonatal outcomes with the severity of preeclampsia, including LBW and preterm births.

Limitations

Despite the valuable insights gained from this research, several limitations must be acknowledged to provide a comprehensive understanding of the findings and their implications. While the study included 74 participants, the relatively small sample size may limit the generalizability of the findings. Our study did not fully account for all potential confounding variables. While adjustments were made for some known confounders, such as maternal age and parity, other important factors like nutritional status and genetic predispositions were not adequately controlled. Additionally, the study's cross-sectional nature limited the ability to assess long-term outcomes. The focus was primarily on immediate and short-term maternal and neonatal outcomes.

## Conclusions

The results in this study indicate that the hematological parameters, which include the platelet count, bleeding time, clotting time, INR, APTT, PT, fibrinogen levels, ALP, D-dimer, and FDP levels, are significantly altered among preeclamptic patients. This warrants an early identification and estimation of coagulation in patients suffering from preeclampsia to reduce maternal and fetal morbidities. Additionally, it also emphasizes a critical requirement for improved antenatal care, and therapeutic initiatives should be tailored to enhance the outcome of pregnant mothers with hypertensive disorders of pregnancy together with the newborn either through induction of labor or C-section.
